# Associations of geriatric nutrition risk index and other nutritional risk-related indexes with sarcopenia presence and their value in sarcopenia diagnosis

**DOI:** 10.1186/s12877-022-03036-0

**Published:** 2022-04-15

**Authors:** Qiao Xiang, Yuxiao Li, Xin Xia, Chuanyao Deng, Xiaochu Wu, Lisha Hou, Jirong Yue, Birong Dong

**Affiliations:** 1 Department of Geriatrics, West China Hospital of Sichuan University, 37 GuoXue Lane, Chengdu, Sichuan 610041 People’s Republic of China; 2 National Clinical Research Center for Geriatrics, West China Hospital of Sichuan University, Chengdu, Sichuan China

## Abstract

**Objective:**

Standard modalities recommended for sarcopenia diagnosis may be unavailable in primary care settings. We aimed to comprehensively evaluate and compare associations of some better popularized nutritional risk-related indexes with sarcopenia presence and their value in sarcopenia diagnosis in community-dwelling middle-aged and elderly adults, including geriatric nutrition risk index (GNRI), albumin (ALB), calf circumference (CC), mid-arm circumference (MAC), triceps skinfold thickness (TST) and body mass index (BMI).

**Methods:**

Based on the West China Health and Aging Trend study, the current study included participants aged 50 or older who were recruited in 2018. Sarcopenia-related assessment and diagnosis were in line with Asian Working Group for Sarcopenia 2019. For each single index, we assessed its association with sarcopenia presence by univariate and multivariate logistic regression analysis; we also computed diagnostic measures including the area under the receiver operating characteristic curve (AUC) and sensitivity, specificity, accuracy at the optimal cut-off value determined according to Youden’s index.

**Results:**

A total of 3829 subjects were included, consisting of 516 and 3313 subjects in the sarcopenia and non-sarcopenia groups, respectively. Regarding the risk for sarcopenia presence, the fully adjusted odds ratios of GNRI, ALB, CC, MAC, TST and BMI per standard deviation decrease were 2.95 (95% CI 2.51–3.47, *P* < 0.001), 1.01 (95% CI 0.90–1.15, *P* = 0.816), 4.56 (95% CI 3.82–5.44, *P* < 0.001), 4.24 (95% CI 3.56–5.05, *P* < 0.001), 1.67 (95% CI 1.92–1.45, *P* < 0.001) and 4.09 (95% CI 3.41–4.91, *P* < 0.001), respectively. Regarding the value in sarcopenia diagnosis in the entire study population, their AUCs could be ordered as MAC (0.85, 95% CI 0.83–0.86) > GNRI (0.80, 95% CI 0.78–0.82), CC (0.83, 95% CI 0.81–0.85), BMI (0.81, 95% CI 0.79–0.83) > TST (0.72, 95% CI 0.70–0.74) > ALB (0.62, 95% CI 0.60–0.65). At the relevant optimal cut-off values, the sensitivity was the highest for CC (0.83, 95% CI 0.80–0.87) and MAC (0.80, 95% CI 0.77–0.84), while GNRI showed the highest specificity (0.79, 95% CI 0.78–0.81) and accuracy (0.78, 95% 0.76–0.79).

**Conclusion:**

Overall diagnostic performance was the best for MAC, followed by GNRI, CC, BMI, and the worst for TST, ALB in distinguishing sarcopenia from non-sarcopenia in middle-aged and elderly adults in community-based settings. CC or MAC might do better in reducing missed diagnosis, while GNRI was superior in reducing misdiagnosis.

**Supplementary Information:**

The online version contains supplementary material available at 10.1186/s12877-022-03036-0.

## Introduction

Sarcopenia, characterized by progressive decline in skeletal muscle mass and function, is a common geriatric syndrome with still evolving and controversial definitions or diagnostic criteria worldwide [1–4]. When defined as age-related loss of skeletal muscle mass plus loss of muscle strength and/or reduced physical performance by the Asian Working Group for Sarcopenia (AWGS) in 2014 [5], sarcopenia showed a prevalence of 5.5–25.7% in Asian countries [6–8], and this original definition was retained in the latest AWGS consensus [9]. Sarcopenia is closely associated with many adverse outcomes in elderly people, including falls, mobility impairment, frailty, physical disability and death [10–12], which attaches great importance to its early detection and intervention.

Either dual-energy X-ray absorptiometry (DXA) or multifrequency bioelectrical impedance analysis (BIA) is recommended by AWGS 2019 to measure muscle mass for sarcopenia diagnosis [9]. However, the two modalities may be unavailable in some primary care settings without advanced diagnostic equipment, calling for easier, less costly and better popularized methods to assist in sarcopenia identification.

As a multifactorial condition with complex mechanisms, sarcopenia not only naturally occurs with aging but can also be caused by various factors, including malnutrition. Malnutrition may often overlap with sarcopenia, highlighting the potential diagnostic value of nutritional risk-related indexes in sarcopenia diagnosis [4, 13, 14]. Simple and cost-effective tools commonly adopted in nutritional risk assessment include screening scales such as the Mini Nutritional Assessment (MNA) or Short-Form MNA (MNA-SF) [15, 16], laboratory indexes such as albumin (ALB) [17], and anthropometric indexes such as calf circumference (CC), mid-arm circumference (MAC), triceps skinfold thickness (TST), body mass index (BMI) [18, 19]. Relationships between the indexes and sarcopenia have been investigated in some studies, but evidence on value and superiorities of the indexes in sarcopenia diagnosis is still limited or disputable [20–25]. Another index, the geriatric nutrition risk index (GNRI), which simultaneously takes ALB, weight and height into consideration, has been suggested as a cost-effective tool in the assessment of nutritional status [26], which has the advantage of more objectivity over questionnaire-based assessments such as the MNA or MNA-SF [15, 16]. On the other hand, sarcopenia is a key phenotypic feature of cachexia [27], which is a complex syndrome reflected in various pathological conditions including malignancies particularly [28, 29], while prognostic value of GNRI has also been reported in several types of cancer [30, 31], suggesting a potential relationship between GNRI and sarcopenia or cachexia. A few studies have investigated the value of GNRI in muscle function-related evaluation and prediction [32–38]. However, some of the previous studies were conducted in people under special conditions, such as male cardiac elderly patients [32] and hemodialysis patients [33, 34]; or the outcome assessments in some studies were only muscle mass, muscle volume indicated by lean mass index (LMI), handgrip strength or physical performance indicated by gait speed without direct reference to sarcopenia [32, 35–37]. One study evaluated the ability of GNRI to identify sarcopenia, but it was conducted in European hospitalized patients according to the European Working Group on Sarcopenia in Older Persons (EWGSOP) definition [38]. No research has thus far focused on the capacity of GNRI to detect older adults with sarcopenia using the Asian criteria in community-based settings, and diagnostic value of the above nutritional risk-related indexes, including GNRI, has not been comprehensively compared.

In this study, we aimed to use data from the West China Health and Aging Trend (WCHAT) study to investigate and compare associations of different nutritional risk-related indexes (especially GNRI) with sarcopenia presence and their value in sarcopenia diagnosis in community-dwelling middle-aged and elderly adults according to the latest AWGS consensus.

## Methods

### Study design and population

The ongoing West China Health and Aging Trend (WCHAT) study, launched in 2018 and registered on the Chinese Clinical Trial Registry (ChiCTR1800018895), is a cohort study designed to explore factors related to healthy aging based on a multiethnic and community-dwelling elderly population in western China. In 2018, people aged 50 or older with a life expectancy of over 6 months and over 3 years of residence in the same region could be recruited into the WCHAT study [39]. Informed consent was obtained from every participant prior to study initiation. The study was approved by the Ethical Committee of Sichuan University West China Hospital (reference: 2017–445) and adhered to the principles of the Declaration of Helsinki.

A total of 7536 Chinese people aged 50 or older from multiethnic groups (Han, Tibetan, Qiang, Yi and other minorities) and 4 provinces (Yunnan, Guizhou, Sichuan, Xinjiang) were initially enrolled in the WCHAT study [39]. We extracted baseline data in 2018 to perform our analysis with exclusion criteria as follows: 1) Participants with unavailable, insufficient or missing information that is necessary for sarcopenia-related diagnosis according to recommendations; 2) Participants with unavailable, insufficient or missing data on height, weight, serum ALB, CC, TST and MAC.

### Data collection

We extracted data regarding the following aspects.

Demographic data: sex, age, ethnicity, history of smoking, history of alcohol consumption, marital status and number of comorbidities.

Questionnaire-based data: results of the Short Portable Mental Status Questionnaire (SPMSQ) [40], Barthel Index for Activities of Daily Living (ADL) [41], Lawton Instrumental ADL (IADL) Scale [42], Generalized Anxiety Disorder 7-item (GAD-7) scale [43] and 15-item Geriatric Depression Scale (GDS-15), [44].

Laboratory data: results of the blood biochemical test, including serum levels of ALB, alanine transaminase (ALT), creatinine (CREA), glucose (GLU), triglyceride (TG), total cholesterol (TC), etc.; results of the blood routine test, including counts of white blood cell (WBC), red blood cell (RBC), hemoglobin, platetlet, etc.; blood levels of thyroid stimulating hormone, free triiodothyroinine (FT3), free throxine (FT4), fasting insulin (INS), cortisol and Vitamin D (VitD) (See the full list of variables measured and analyzed in Supplementary Table 1).

Other data: anthropometric data including height, weight, CC, TST and MAC; handgrip strength; physical performance-related data (gait speed in the 4-m walking test, time consumed in the 5-time chair stand test [9]); the appendicular skeletal muscle mass (ASM) index (ASMI).

Demographic and questionnaire-based data were collected through face-to-face interviews by medical students or volunteers who had received relevant training. Number of comorbidities referred to the total number of self-reported chronic diseases among hypertension, coronary heart disease, chronic obstructive pulmonary disease, diabetes, osteoarthrosis, digestive disease and renal disease. The SPMSQ was used to determine the presence and degree of organic brain deficit, with ≥5 errors considered moderate to severe cognitive impairment [40]. ADL or IADL each represents daily self-care activities to support fundamental functioning or independent living [45, 46], with ADL or IADL impairment indicated by a total Barthel Index score of < 100 or Lawton IADL Scale score of < 14, respectively [41, 42]. The GAD-7 scale was used to screen and grade the severity of generalized anxiety disorder, with a total score of ≥10 referring to moderate to severe anxiety [43]. The GDS-15 was used to identify depression, and moderate to severe depression was indicated by a score of ≥9 [44].

Laboratory data were obtained from fasting blood samples taken in the early morning. GNRI was calculated according to the previously proposed equation: GNRI = 1.489 × serum albumin (g/L) + 41.7 × present weight/ideal weight (kg). The ideal weight was derived from the Lorentz formula as follows: ideal weight for women = 0.60 × height (cm) – 40, ideal weight for men = 0.75 × height (cm) – 62.5, and a present weight/ideal weight ratio is set to 1 if it is no less than 1 [47]. Using the GNRI cut-off values suggested by Cereda et al., all the study participants were classified into 3 subgroups indicating different risk levels of nutritional-related complications: GNRI > 98, no risk; GNRI 92–98, low risk; GNRI < 92, major/moderate risk [47].

Measurements of anthropometrics, handgrip strength, physical performance and ASMI were performed by well-experienced inspectors. CC, TST and MAC were measured twice, with the average value of two measurements used for analysis. BMI was calculated as weight (kg) divided by the square of height (m^2^). Handgrip strength was measured on the dominant hand twice by the myometer EH101 (Camry, Zhongshan, China), with the maximum recorded for analysis. ASMI was obtained from bioelectrical impedance analysis (BIA) using Inbody 770 (BioSpace, Seoul, Korea).

### Assessment and diagnosis related to sarcopenia

According to AWGS 2019 and available data on our study population, possible sarcopenia was defined by low muscle strength (a handgrip strength of < 28 kg for men and < 18 kg for women) or low physical performance (a gait speed of < 1.0 m/s in the 4-m walking test or a time of ≥12 s in the 5-time chair stand test) regardless of ASMI [9].

A definitive diagnosis of sarcopenia required low ASM (an ASMI of < 7.0 kg/m^2^ for men and < 5.7 kg/m^2^ for women) plus low muscle strength and/or low physical performance [9].

Among patients with confirmed sarcopenia, those showing coexistence of low ASM with both low muscle strength and low physical performance were further referred to as having severe sarcopenia, while the rest were classified as non-severe cases in this study [9].

### Statistical analyses

Continuous variables in normal or skewness distribution were presented as mean and standard deviation (SD) or median and quartile 1 (Q1), quartile 3 (Q3), respectively; categorical variables were presented as number and percentage (%). Data comparison between groups was performed: continuous variables were compared using Student’s t test or Kruskal-Wallis H test for normally or non-normally distributed data; categorical variables were compared using the Chi-square test or Fisher’s exact test.

Correlations between continuous variables were analyzed by Pearson’s correlation coefficient. Univariate or multivariate logistic regression analysis was performed to assess the association of the concerned indexes (GNRI, ALB, CC, MAC, TST and BMI) with sarcopenia indicated by the odds ratio (OR) in the unadjusted or adjusted models, respectively. Model 1 was unadjusted for any factors; model 2 was adjusted for age and sex; model 3 was further adjusted for other variables (shown in the Results section) on the basis of model 2. Variables showing significant differences between groups in the baseline comparison, previously reported to be associated with sarcopenia, or considered to have clinical implications were treated as potential variables to be controlled in model 3. Variance inflation factors (VIFs) of the potential continuous variables and their reciprocal Pearson correlation coefficients were calculated to detect multicollinearity, and variables with a VIF of ≥10 or correlation coefficients of > 0.7 were subsequently excluded from model 3 [48, 49]. We performed separate analyses that treated the concerned indexes as either continuous or categorical variables (categorized into > 98, 92–98 and < 92 for GNRI; categorized into tertiles for ALB, CC, MAC, TST and BMI: low T1, middle T2, high T3) in the models.

We further assessed the diagnostic value of GNRI, ALB, CC, MAC, TST and BMI by constructing the receiver operating characteristic (ROC) curve regarding the following two aspects: to distinguish between sarcopenia and non-sarcopenia in the entire study population and to confirm or exclude a definitive diagnosis in patients with possible sarcopenia. The relevant area under the curve (AUC) was computed and compared as proposed by DeLong et al. [50]. The optimal cut-off value was determined according to Youden’s index, with the corresponding sensitivity, specificity and accuracy at that cut-off value calculated and compared using the McNemar chi-square test.

All statistical analyses were performed using Python (version 3.8.8) and R (version 4.0.3). *P* values < 0.05 were considered statistically significant.

## Results

**Fig. 1:**
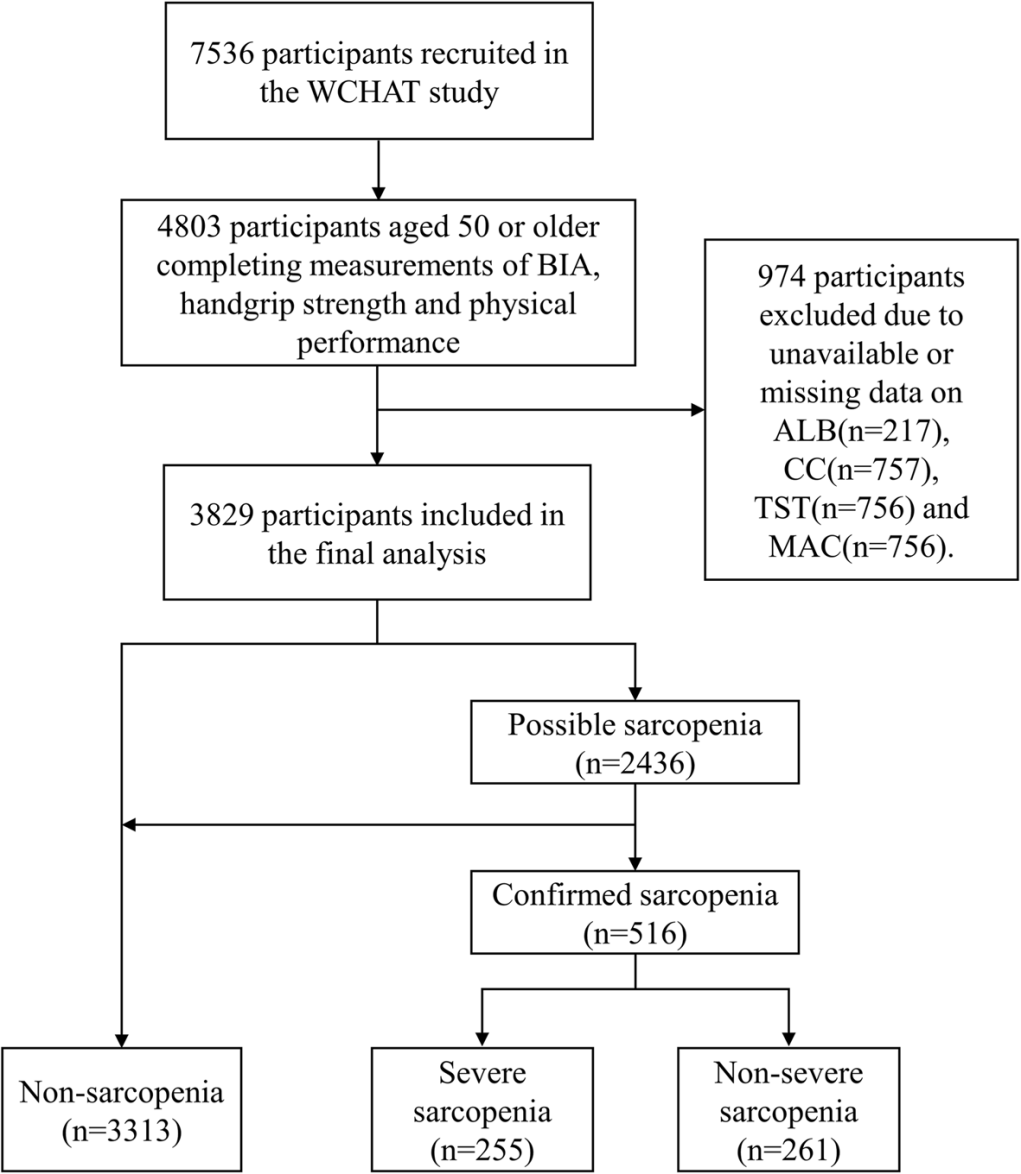
The flowchart of inclusion, exclusion and diagnosis of participants. Abbreviations: WCHAT, West China Health and Aging Trend; BIA, bioelectrical impedance analysis; ALB, albumin; CC, calf circumference; TST, triceps skinfold thickness; MAC, mid-arm circumference

Figure 1 displays the flow path of inclusion, exclusion and diagnosis of participants. A total of 3829 participants were finally included in the study with a median age of 62.0 years and male proportion of 35.9% from different ethnic backgrounds (43.4% for Han, 25.7% for Qiang, 24.4% for Tibetan, 4.9% for Yi and 1.6% for other minority).

The definitive diagnosis of sarcopenia was finally confirmed in 516 cases and excluded in 3313 cases. The prevalence of sarcopenia vs non-sarcopenia was 13.5% vs 86.5% in the entire study population, 21.2% vs 78.8% in all the males and 9.1% vs 90.9% in all the females. Among the 516 patients with sarcopenia, 255 and 261 cases were classified as severe and non-severe, respectively.

A total of 2436 participants were referred to as having possible sarcopenia, consisting of 516 (78.8%) and 1920 (21.2%) cases finally included in the sarcopenia and non-sarcopenia group, respectively.

### Data comparison between groups

#### Sarcopenia vs non-sarcopenia

Compared with non-sarcopenic individuals, patients with sarcopenia were older, showed different ethnic backgrounds and marital status, had higher percentage of men, smokers, ADL or IADL impairment and moderate to severe cognitive impairment; they also showed significantly higher levels of direct bilirubin, CREA, high-density lipoprotein (HDL), absolute neutrophil count, neutrophilic granulocyte percentage, RBC distribution width (RDW)-SD, RDW-coefficient of variation (RDW-CV), mean corpuscular volume, mean corpuscular hemoglobin, FT4 and plasma total cortisol but lower levels of indirect bilirubin, total protein, ALT, GLU, TG, TC, absolute lymphocyte count, lymphocyte percentage, RBC, plateletcrit, mean platelet volume, platelet distribution width, platelet large cell ratio, FT3 INS and VitD (*P* < 0.05). The sarcopenia group exhibited significantly lower levels of GNRI, ALB, CC, MAC, TST and BMI (*P* < 0.05) (Table 1).

**Table 1:** Comparison of baseline data between non-sarcopenia and sarcopenia

	Non-sarcopenia ***n*** = 3313	Sarcopenia ***n*** = 516	***P*** value
**Age**	61.00 [55.00,66.00]	69.00 [63.00,75.00]	< 0.001
**Sex, n (%)**			< 0.001
Male	1084 (32.72%)	292 (56.59%)	
Female	2229 (67.28%)	224 (43.41%)	
**Ethnicity, n (%)**			< 0.001
Han	1401 (42.29%)	261 (50.58%)	
Qiang	902 (27.23%)	80 (15.50%)	
Tibetan	812 (24.51%)	122 (23.64%)	
Yi	145 (4.38%)	44 (8.53%)	
Other minority	53 (1.60%)	9 (1.74%)	
**Marital status, n (%)**			< 0.001
Single	19 (0.57%)	8 (1.55%)	
Married	2827 (85.33%)	392 (75.97%)	
Divorced	53 (1.60%)	10 (1.94%)	
Widowed	414 (12.50%)	106 (20.54%)	
**Smoking, n (%)**	517 (15.61%)	150 (29.07%)	< 0.001
**Drinking alcohol, n (%)**	853 (25.75%)	136 (26.36%)	0.81
**ADL, n (%)**			< 0.001
Normal ADL	3003 (90.64%)	439 (85.08%)	
ADL impairment	310 (9.36%)	77 (14.92%)	
**IADL, n (%)**			< 0.001
Normal IADL	2657 (80.20%)	336 (65.12%)	
IADL impairment	656 (19.80%)	180 (34.88%)	
**Moderate to severe cognitive impairment, n (%)**	415 (12.53%)	108 (20.93%)	< 0.001
**Number of comorbidities, n (%)**			0.13
0	1878 (56.69%)	288 (55.81%)	
1	749 (22.61%)	103 (19.96%)	
≥ 2	686 (20.71%)	125 (24.22%)	
**Moderate to severe anxiety, n (%)**	125 (3.77%)	19 (3.68%)	0.981
**Moderate to severe depression, n (%)**	149 (4.50%)	28 (5.43%)	0.411
**Total bilirubin (umol/L)**	18.10 [14.40,20.30]	18.00 [14.07,20.30]	0.397
**Direct bilirubin (umol/L)**	5.30 [4.20,6.80]	5.50 [4.30,7.30]	0.027
**Indirect bilirubin (umol/L)**	12.50 [10.00,14.40]	12.00 [9.40,14.20]	0.004
**Total protein (g/L)**	71.91 (5.88)	70.61 (5.13)	< 0.001
**Globulin (g/L)**	27.50 (4.98)	27.59 (3.99)	0.653
**ALT (U/L)**	24.00 [18.00,33.00]	19.00 [15.00,26.25]	< 0.001
**AST (U/L)**	26.00 [22.00,32.00]	27.00 [23.00,33.00]	0.11
**CREA (umol/L)**	80.33 (19.06)	83.14 (19.98)	0.003
**Urea (mmol/L)**	5.40 (1.60)	5.50 (1.82)	0.247
**Uric acid (umol/L)**	328.50 (81.68)	329.49 (90.39)	0.815
**GLU (mmol/L)**	5.17 [4.81,5.70]	5.01 [4.67,5.47]	< 0.001
**TG (mmol/L)**	1.45 [1.00,2.16]	1.23 [0.89,1.73]	< 0.001
**TC (mmol/L)**	4.81 (0.92)	4.66 (0.95)	0.001
**HDL (mmol/L)**	1.26 (0.30)	1.34 (0.36)	< 0.001
**LDL (mmol/L)**	2.68 (0.88)	2.65 (0.80)	0.498
**WBC (10**^**9**^**/L)**	5.60 [4.80,6.60]	5.70 [4.80,6.70]	0.308
**Absolute neutrophil count (10**^**9**^**/L)**	3.40 [2.70,4.20]	3.60 [2.70,4.30]	0.034
**Absolute lymphocyte count (10**^**9**^**/L)**	1.70 [1.40,2.10]	1.60 [1.30,2.00]	0.003
**Neutrophilic granulocyte percentage (%)**	60.91 (8.51)	62.33 (9.48)	0.001
**Lymphocyte percentage (%)**	31.72 (7.85)	30.39 (8.63)	0.001
**RBC (10**^**12**^**/L)**	4.93 (0.58)	4.86 (0.62)	0.008
**RDW-SD (fL)**	51.94 (4.38)	53.83 (5.12)	< 0.001
**RDW-CV (%)**	14.63 (0.76)	14.94 (0.94)	< 0.001
**Hemoglobin (g/L)**	148.47 (17.32)	147.77 (18.66)	0.418
**Hematocrit (L/L)**	47.17 (5.56)	47.09 (6.21)	0.775
**Mean corpuscular volume (fL)**	96.00 [93.20,98.90]	96.95 [93.90,99.90]	< 0.001
**Mean corpuscular hemoglobin (pg)**	30.40 [29.40,31.30]	30.50 [29.60,31.60]	0.001
**MCHC (g/L)**	315.00 [311.00,319.00]	315.00 [311.00,319.25]	0.758
**Platetlet (10**^**9**^**/L)**	168.71 (55.92)	168.79 (57.65)	0.975
**Plateletcrit (%)**	0.18 [0.15,0.21]	0.17 [0.14,0.20]	0.032
**Mean platelet volume (fL)**	11.10 [10.00,12.30]	10.70 [9.70,11.90]	< 0.001
**Platelet distribution width (%)**	13.50 [12.20,15.30]	13.00 [11.70,14.70]	< 0.001
**Platelet large cell ratio (%)**	34.90 [27.50,42.30]	31.65 [24.68,40.23]	< 0.001
**Thyroid stimulating hormone (mU/L)**	2.82 [1.84,4.26]	2.66 [1.73,4.23]	0.172
**FT3 (pmol/l)**	4.49 [4.10,4.93]	4.36 [3.97,4.80]	< 0.001
**FT4 (pmol/l)**	17.82 [16.14,19.53]	18.42 [16.36,20.50]	< 0.001
**INS (uU/ml)**	7.18 [4.89,10.45]	4.53 [3.01,6.66]	< 0.001
**Plasma total cortisol (nmol/L)**	343.07 (147.67)	383.36 (136.04)	< 0.001
**VitD (ng/ml)**	19.29 (6.24)	18.58 (6.37)	0.019
**GNRI**	114.53 (8.19)	104.96 (8.17)	< 0.001
**ALB (g/L)**	44.41 (2.96)	43.03 (3.33)	< 0.001
**CC (cm)**	35.32 (2.94)	31.70 (2.77)	< 0.001
**MAC (cm)**	29.43 (3.02)	25.51 (2.62)	< 0.001
**TST (cm)**	25.01 (8.29)	18.55 (7.27)	< 0.001
**BMI (kg/m**^**2**^**)**	25.79 (3.54)	21.91 (3.01)	< 0.001
**ASMI (kg/m**^**2**^**)**	6.76 (0.89)	5.79 (0.81)	< 0.001
**Handgrip strength (kg)**	22.71 (8.70)	18.23 (6.86)	< 0.001
**Time consumed in the 4-m walking test (s)**	5.02 (1.96)	5.93 (2.93)	< 0.001
**Time consumed in the 5-time chair stand test (s)**	11.32 (3.14)	13.28 (3.89)	< 0.001

*Note*: data were presented as mean (standard deviation), median [quartile 1, quartile 3] or n (%) as appropriate

*Abbreviations*: *ADL* Activities of Daily Living, *IADL* Instrumental ADL, *ALT* alanine transaminase, *AST* aspartate aminotransferase, *CREA* creatinine, *GLU* glucose, *TG* triglyceride, *TC* total cholesterol, *HDL* high-density lipoprotein, *LDL* low-density lipoprotein, *WBC* white blood cell, *RBC* red blood cell, *RDW-SD* RBC distribution width-standard deviation, *RDW-CV* RBC distribution width-coefficient of variation, *MCHC* mean corpuscular hemoglobin concentration, *FT3* free triiodothyroinine, *FT4* free throxine, *INS* fasting insulin, *VitD* Vitamin D, *GNRI* geriatric nutrition risk index, *ALB* albumin, *CC* calf circumference, *MAC* mid-arm circumference, *TST* triceps skinfold thickness, *BMI* body mass index, *ASMI* appendicular skeletal muscle mass index

#### Severe vs non-severe sarcopenia

Compared with patients with non-severe sarcopenia, severely sarcopenic patients were older, showed different marital status, and had a higher percentage of ADL or IADL impairment as well as multiple comorbidities (≥2); they also showed significantly higher levels of HDL and RDW-CV but lower levels of ALT and FT3 (*P* < 0.05). CC was significantly lower in the severe than non-severe sarcopenia group (*P* < 0.05), while GNRI, ALB, MAC, TST and BMI did not differ between the two groups (Supplementary Table 2).

### Associations of the concerned indexes with muscle mass, muscle strength, physical performance and sarcopenia presence

**Fig. 2:**
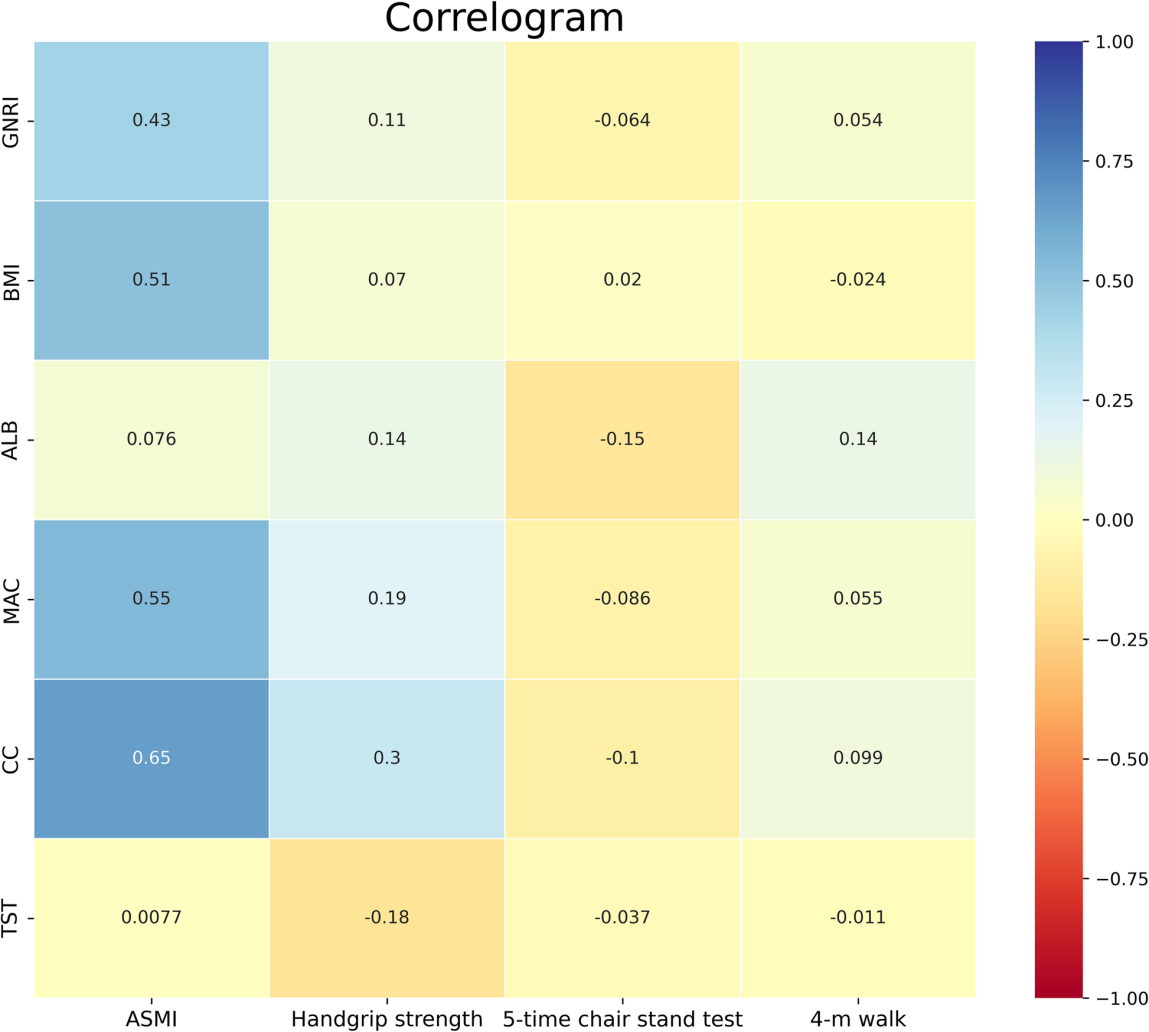
The correlogram showing correlations of the concerned diagnostic indexes (vertical) with muscle mass, muscle strength and physical performance (horizontal). Muscle mass was indicated by ASMI; muscle strength was indicated by handgrip strength; physical performance was indicated by 4-m walk and 5-time chair stand test. The value in each module was the corresponding Pearson’s correlation coefficient between relevant variables. The strength of correlation was represented by module color with a gradient from blue (weakest) to red (strongest). Abbreviations: GNRI, geriatric nutrition risk index; ALB, albumin; CC, calf circumference; MAC, mid-arm circumference; TST, triceps skinfold thickness; BMI, body mass index; ASMI, appendicular skeletal muscle mass index; 4-m walk, gait speed in the 4-m walking test; 5-time chair stand test, time consumed in the 5-time chair stand test

In the entire study population, GNRI, ALB, CC, and MAC each positively correlated with ASMI, handgrip strength and gait speed, while they negatively correlated with time consumed in the 5-time chair stand test. BMI was only positively correlated with ASMI and handgrip strength, while TST was only negatively correlated with handgrip strength and time consumed in the 5-time chair stand test (*P* < 0.05) (Fig. 2) (Table 2).

**Table 2:** Correlations of the concerned indexes with muscle mass, muscle strength and physical performance

Concerned indexes	Muscle mass	Muscle strength	Physical performance	
	ASMI (kg/m^**2**^)	Handgrip strength (kg)	Gait speed in the 4-m walking test (s)	Time consumed in the 5-time chair stand test (s)
**GNRI**	0.43 (*P* < 0.001)	0.11 (*P* < 0.001)	0.05 (*P* = 0.001)	−0.06 (*P* < 0.001)
**ALB (g/L)**	0.08 (*P* < 0.001)	0.14 (*P* < 0.001)	0.14 (*P* < 0.001)	−0.15 (*P* < 0.001)
**CC (cm)**	0.65 (*P* < 0.001)	0.30 (*P* < 0.001)	0.10 (*P* < 0.001)	−0.10 (*P* < 0.001)
**MAC (cm)**	0.55 (*P* < 0.001)	0.19 (*P* < 0.001)	0.06 (*P* = 0.001)	−0.09 (*P* < 0.001)
**TST (cm)**	0.01 (*P* = 0.633)	−0.18 (*P* < 0.001)	−0.01 (*P* = 0.633)	− 0.04 (*P* = 0.021)
**BMI (kg/m**^**2**^**)**	0.51 (*P* < 0.001)	0.07 (*P* < 0.001)	−0.02 (*P* = 0.142)	0.02 (*P* = 0.221)

*Note*: data were presented as the Pearson’s correlation coefficient (*P* value)

*Abbreviations*: *GNRI* geriatric nutrition risk index, *ALB* albumin, *CC* calf circumference, *MAC* mid-arm circumference, *TST* triceps skinfold thickness, *BMI* body mass index, *ASMI* appendicular skeletal muscle mass index

We selected the following variables to be corrected for in model 3 as previously described (Supplementary Table 3, Supplementary Figure 1): age, sex, ethnicity (compared to Han), marriage status (compared to being married), smoking, drinking alcohol, ADL impairment, IADL impairment, moderate to severe cognitive impairment, number of comorbidities (compared to no comorbidity), moderate to severe anxiety, moderate to severe depression, indirect bilirubin, ALT, CREA, GLU, TG, TC, HDL, WBC, absolute neutrophil count, absolute lymphocyte count, RBC, RDW-CV, plateletcrit, platelet distribution width, FT3, FT4, INS, plasma total cortisol and VitD.

When treated as continuous variables, lower levels of GNRI, CC, MAC, TST and BMI were all associated with a higher prevalence of sarcopenia both in the unadjusted and adjusted models. After full adjustment for other variables (model 3), we found that with each unit or SD reduction in GNRI, CC, MAC, TST and BMI, the risk for sarcopenia presence increased by 13% or 1.95 times, 61% or 3.56 times, 56% or 3.24 times, 6% or 67 and 46% or 3.09 times, respectively. Lower ALB levels were significantly associated with a higher prevalence of sarcopenia in the unadjusted model or when adjusted for age and sex, but the association disappeared after fully adjusting for other variables in model 3 (Table 3).

**Table 3:** Univariate and multivariate logistic regression analysis on associations of the concerned indexes with sarcopenia presence

Concerned indexes	Model 1^**a**^ (Unadjusted)	Model 2^**b**^	Model 3^**c**^
	OR (95% CI), ***P*** value	OR (95% CI), ***P*** value	OR (95% CI), ***P*** value
**GNRI**			
per unit decrease	1.16 (1.14–1.18), < 0.001	1.14 (1.12–1.16), < 0.001	1.13 (1.11–1.15), < 0.001
per SD decrease	3.67 (3.24–4.15), < 0.001	3.16 (2.77–3.59), < 0.001	2.95 (2.51–3.47), < 0.001
92-98^d^	10.48 (7.29–15.08), < 0.001	7.60 (5.02–11.49), < 0.001	4.18 (2.64–6.61), < 0.001
< 92^d^	21.64 (10.03–46.68), < 0.001	12.78 (5.60–29.16), < 0.001	6.43 (2.57–16.09), < 0.001
**ALB (g/L)**			
per unit decrease	1.16 (1.13–1.20), < 0.001	1.10 (1.06–1.13), < 0.001	1.00 (0.97–1.05), 0.816
per SD decrease	1.58 (1.44–1.74), < 0.001	1.33 (1.20–1.47), < 0.001	1.01 (0.90–1.15), 0.816
T2 (43.0, 45.4]^e^	1.10 (0.85–1.43), 0.478	0.97 (0.74–1.28), 0.825	0.91 (0.68–1.22), 0.528
T1(≤43.0)^e^	2.40 (1.91–3.02), < 0.001	1.63 (1.27–2.09), < 0.001	0.81 (0.60–1.10), 0.175
**CC (cm)**			
per unit decrease	1.59 (1.53–1.66), < 0.001	1.69 (1.61–1.78), < 0.001	1.61 (1.53–1.71), < 0.001
per SD decrease	4.38 (3.82–5.02), < 0.001	5.31 (4.51–6.26), < 0.001	4.56 (3.82–5.44), < 0.001
T2 (33.5, 36.1]^f^	3.31 (2.16–5.07), < 0.001	3.46 (2.23–5.37), < 0.001	2.91 (1.85–4.57), < 0.001
T1 (≤33.5)^f^	18.82 (12.79–27.71), < 0.001	23.63 (15.69–35.59), < 0.001	16.75 (10.89–25.77), < 0.001
**MAC (cm)**			
per unit decrease	1.61 (1.54–1.68), < 0.001	1.58 (1.51–1.66), < 0.001	1.56 (1.48–1.64), < 0.001
per SD decrease	4.72 (4.11–5.42), < 0.001	4.49 (5.22–3.85), < 0.001	4.24 (3.56–5.05), < 0.001
T2 (27.5, 30.3]^g^	4.65 (2.77–7.81), < 0.001	4.17 (2.46–7.05), < 0.001	3.72 (2.17–6.38), < 0.001
T1 (≤27.5)^g^	32.68 (20.23–52.78), < 0.001	28.12 (17.24–45.84), < 0.001	22.75 (13.60–38.04), < 0.001
**TST (cm)**			
per unit decrease	1.11 (1.10–1.12), < 0.001	1.09 (1.08–1.11), < 0.001	1.06 (1.05–1.08), < 0.001
per SD decrease	2.42 (2.17–2.71), < 0.001	2.12 (1.87–2.41), < 0.001	1.67 (1.92–1.45), < 0.001
T2 (20.8, 27.6]^h^	2.46 (1.80–3.37), < 0.001	1.98 (1.43–2.75), < 0.001	1.73 (1.22–2.44), < 0.001
T1 (≤20.8)^h^	6.61 (4.95–8.81), < 0.001	4.42 (3.23–6.05), < 0.001	2.99 (2.14–4.19), < 0.001
**BMI (kg/m**^**2**^**)**			
per unit decrease	1.48 (1.43–1.54), < 0.001	1.48 (1.42–1.54), < 0.001	1.46 (1.39–1.53), < 0.001
per SD decrease	4.31 (3.74–4.97), < 0.001	4.26 (3.65–4.97), < 0.001	4.09 (3.41–4.91), < 0.001
T2 (23.5, 26.7] ^i^	2.97 (1.99–4.43), < 0.001	2.91 (1.93–4.39), < 0.001	2.96 (1.93–4.55), < 0.001
T1(≤23.5)^i^	15.83 (11.03–22.71), < 0.001	15.85 (10.89–23.06), < 0.001	13.62 (8.98–20.66), < 0.001

*Note*: ^a^ Model 1 was unadjusted for any factors

^b^ Model 2 was adjusted for age and sex

^c^ Model 3 was adjusted for age, sex, ethnicity (compared to Han), marriage status (compared to being married), smoking, drinking alcohol, ADL impairment, IADL impairment, moderate to severe cognitive impairment, number of comorbidities (compared to no comorbidity), moderate to severe anxiety, moderate to severe depression, indirect bilirubin, ALT, CREA, GLU, TG, TC, HDL, WBC, absolute neutrophil count, absolute lymphocyte count, RBC, RDW-CV, plateletcrit, platelet distribution width, FT3, FT4, INS, plasma total cortisol and VitD

^d^ Compared to GNRI > 98 as reference

^e^ Compared to ALB in the T3 group (> 45.4 g/L) as reference

^f^ Compared to CC in the T3 group (> 36.1 cm) as reference

^g^ Compared to MAC in the T3 group (> 30.3 cm) as reference

^h^ Compared to TST in the T3 group (> 27.6 cm) as reference

^i^ Compared to BMI in the T3 group (> 26.7 kg/m^2^) as reference

*Abbreviations*: *OR* odds ratio, *CI* confidence interval, *T1* tertile 1 (low), *T2* tertile 2 (middle), *T3* tertile 3 (high), *GNRI* geriatric nutrition risk index, *ALB* albumin, *CC* calf circumference, *MAC* mid-arm circumference, *TST* triceps skinfold thickness, *BMI* body mass index, *ADL* Activities of Daily Living, *IADL* Instrumental ADL, *ALT* alanine transaminase, *CREA* creatinine, *GLU* glucose, *TG* triglyceride, *TC* total cholesterol, *HDL* high-density lipoprotein, *WBC* white blood cell, *RBC* red blood cell, *RDW-CV* RBC distribution width-coefficient of variation, *FT3* free triiodothyroinine, *FT4* free throxine, *INS* fasting insulin, *VitD* Vitamin D

When we treated the concerned indexes as categorical variables and used the relevant group at the highest level as reference, similar results were yielded both before and after adjustment. In the ultimately adjusted model (model 3), the risk for sarcopenia for individuals with GNRI < 92 (GNRI-defined major/moderate nutritional risk) or in the T1 groups of CC, MAC, TST and BMI was 6.43, 16.75, 22.75, 2.99 and 13.62 times as much as that for those with GNRI > 98 (no nutritional risk defined by GNRI) or in the respective T3 groups. ALB at T1 instead of T2 level was significantly associated with a higher prevalence of sarcopenia than that at T3 level in the unadjusted model or when adjusted for age and sex, while the association disappeared after fully adjusting for other variables in model 3 (Table 3).

### Diagnostic assessment on the concerned indexes

#### To distinguish between sarcopenia and non-sarcopenia in the entire study population

**Fig. 3:**
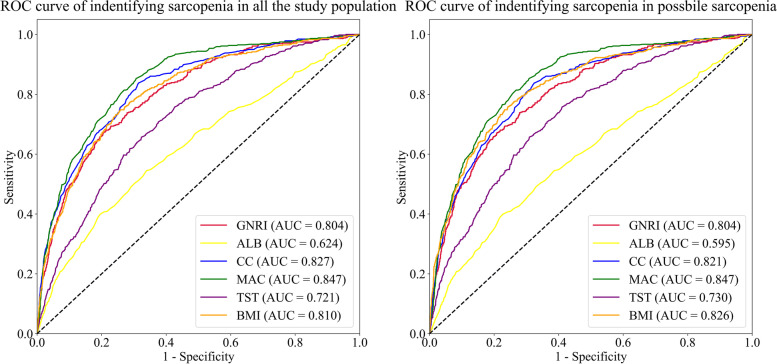
ROC curves of the concerned indexes for distinguishing between sarcopenia and non-sarcopenia in the entire study population (Fig. 3a) and in possible sarcopenia (Fig. 3b). Abbreviations: ROC, receiver operating characteristic; AUC, area under the ROC curve; GNRI, geriatric nutrition risk index; ALB, albumin; CC, calf circumference; MAC, mid-arm circumference; TST, triceps skinfold thickness; BMI, body mass index

The AUCs of GNRI, ALB, CC, MAC, TST and BMI to identify sarcopenia in the entire study population were 0.80 (95% CI 0.78–0.82), 0.62 (95% CI 0.60–0.65), 0.83 (95% CI 0.81–0.85), 0.85 (95% CI 0.83–0.86), 0.72 (95% CI 0.70–0.74) and 0.81 (95% CI 0.79–0.83), respectively (*P*<0.05) (Fig. 3a, Table 4), which could be ordered from largest to smallest as follows: MAC > GNRI, CC, BMI (not significantly different) > TST > ALB (*P*<0.05 in pairwise comparison, Supplementary Table 4).

**Table 4:** Diagnostic value of the concerned indexes for distinguishing between sarcopenia and non-sarcopenia

	AUC (95% CI)	Cut-off	Sensitivity (95% CI)	Specificity (95% CI)	Accuracy (95% CI)
**In the entire study population (*N*** **= 3829)**					
GNRI	0.80 (0.78–0.82)	108.1	0.67 (0.63–0.71)	0.79 (0.78–0.81)	0.78 (0.76–0.79)
ALB	0.62 (0.60–0.65)	43.2 g/L	0.53 (0.48–0.57)	0.67 (0.66–0.69)	0.65 (0.64–0.67)
CC	0.83 (0.81–0.85)	34.0 cm	0.83 (0.80–0.87)	0.69 (0.67–0.7)	0.71 (0.69–0.72)
MAC	0.85 (0.83–0.86)	27.5 cm	0.80 (0.77–0.84)	0.74 (0.73–0.76)	0.75 (0.74–0.76)
TST	0.72 (0.70–0.74)	21.4 cm	0.65 (0.61–0.69)	0.68 (0.66–0.69)	0.67 (0.66–0.69)
BMI	0.81 (0.79–0.83)	23.4 kg/m^2^	0.75 (0.71–0.78)	0.74 (0.73–0.76)	0.74 (0.73–0.76)
**In possible sarcopenia (*N*** **= 2436)**					
GNRI	0.80 (0.78–0.83)	108.1	0.67 (0.63–0.71)	0.79 (0.77–0.81)	0.77 (0.75–0.78)
ALB	0.60 (0.57–0.62)	42.0 g/L	0.39 (0.35–0.43)	0.78 (0.76–0.80)	0.70 (0.68–0.72)
CC	0.82 (0.80–0.84)	34.0 cm	0.83 (0.80–0.87)	0.68 (0.66–0.70)	0.71 (0.69–0.73)
MAC	0.85 (0.83–0.87)	27.5 cm	0.80 (0.77–0.84)	0.74 (0.72–0.76)	0.76 (0.74–0.77)
TST	0.73 (0.71–0.75)	21.4 cm	0.65 (0.61–0.69)	0.69 (0.67–0.71)	0.68 (0.66–0.70)
BMI	0.83 (0.81–0.85)	23.4 kg/m^2^	0.75 (0.71–0.78)	0.77 (0.75–0.79)	0.76 (0.75–0.78)

*Note*: The AUCs of all the concerned indexes were significantly larger than the area under the diagnostic reference line (*P*<0.05). Sensitivity, specificity and accuracy all referred to the corresponding diagnostic measures at the optimal cut-off values

*Abbreviations*: *AUC* area under the receiver operating characteristic curve, *CI* confidence interval, *Cut-off* the optimal cut-off value determined according to Youden’s index, *GNRI* geriatric nutrition risk index, *ALB* albumin, *CC* calf circumference, *MAC* mid-arm circumference, *TST* triceps skinfold thickness, *BMI* body mass index

The optimal cut-off values for GNRI, ALB, CC, MAC, TST and BMI to identify sarcopenia in the entire study population were 108.1, 43.2 g/L, 34.0 cm, 27.5 cm, 21.4 cm and 23.4 kg/m^2^, respectively. Their diagnostic measures at the corresponding cut-off values from highest to lowest could be ordered as follows: for sensitivities, CC (0.83, 95% CI 0.80–0.87), MAC (0.80, 95% CI 0.77–0.84) > BMI (0.75, 95% CI 0.71–0.78) > GNRI (0.67, 95% CI 0.63–0.71), TST (0.65, 95% CI 0.61–0.69) > ALB (0.53, 95% CI 0.48–0.57); for specificities, GNRI (0.79, 95% CI 0.78–0.81) > MAC (0.74, 95% CI 0.73–0.76), BMI (0.74, 95% CI 0.73–0.76) > ALB (0.67, 95% CI 0.66–0.69), CC (0.69, 95% CI 0.67–0.70), TST (0.68, 95% CI 0.66–0.69); for accuracies, GNRI (0.78, 95% 0.76–0.79) > MAC (0.75, 95% CI 0.74–0.76), BMI (0.74, 95% CI 0.73–0.76) > CC (0.71, 95% CI 0.69–0.72) > ALB (0.65, 95% CI 0.64–0.67), TST (0.67, 95% CI 0.66–0.69) (*P* < 0.05 in the McNemar chi-square test, Supplementary Table 4) (Table 4).

#### To confirm or exclude a definitive diagnosis in patients with possible sarcopenia

The AUCs of all the concerned indexes to identify sarcopenia in possible sarcopenia corresponded closely to those in all the study population (*P* < 0.05) (Fig. 3b, Table 4), and they were ranked in almost the same order of value except that BMI (AUC = 0.83, 95% CI 0.81–0.85) had a significantly larger AUC than GNRI (AUC = 0.80, 95% CI 0.78–0.83) (*P*<0.05 in pairwise comparison, Supplementary Table 5).

Compared with in the entire study population, the concerned indexes also had identical or similar optimal cut-off values and relevant sensitivities, specificities or accuracies as well as their ranking orders when used in possible sarcopenia except for ALB. At the optimal cut-off value of 42.0 g/L, the sensitivity of ALB decreased to 0.39 (95% CI 0.35–0.43), while its specificity increased to 0.78 (95% CI 0.76–0.80, not significantly different from GNRI but higher than MAC or BMI) and its accuracy increased to 0.70 (95% CI 0.68–0.72, higher than TST but lower than CC) (Table 4) (*P* < 0.05 in the McNemar chi-square test, Supplementary Table 5).

## Discussion

Based on the WCHAT study, we elucidated associations between different nutritional risk-related indexes and the risk for sarcopenia presence in a relatively large sample size, especially focused on GNRI. Besides, we assessed value of the indexes in sarcopenia diagnosis and made comparison to show their superiority or inferiority in different diagnostic aspects. To the best of our knowledge, this study provides us with the most comprehensive understanding of the diagnostic utility of the indexes commonly adopted in nutritional assessment for sarcopenia identification in the Chinese population. This study also revealed for the first time the capacity of GNRI to detect middle-aged and elderly adults with sarcopenia in Asian community-based settings according to the latest AWGS consensus.

Some research has previously investigated the relationship between GNRI and muscle function [32–38]. Significant correlations of GNRI with handgrip strength, arm muscle area and strength for centimeter of muscle area were revealed in European institutionalized elderly people [36]; another study conducted in Chinese elderly individuals who received health check-ups also showed a close association between lower GNRI and low muscle mass [35]; a Japanese study found GNRI to be a useful predictor of physical performance indicated by gait speed in male cardiac patients [32]; in patients receiving hemodialysis, GNRI was found to correlate with both muscle volume or strength indicated by handgrip strength [34] or LMI [37] and the risk for sarcopenia [33]. Consistently, our study further supported associations of GNRI with muscle mass, muscle strength, physical performance and the risk for sarcopenia, which overcame limitation in previous studies, including absence of muscle function assessment, unavailability of the sarcopenia definition or restriction on population selection. A recent study by Takahashi F et al. [51] demonstrated the association between low GNRI and sarcopenia presence in outpatients with type 2 diabetes mellitus (T2DM), with sarcopenia defined according to AWGS 2019 [9] but only based on low muscle mass plus low muscle strength in the absence of physical performance evaluation. Therefore, there was possibility that some sarcopenic patients in that study might be misclassified as non-sarcopenic if they had low muscle mass, low physical performance but normal muscle strength, which could weaken validity of its conclusion, as discussed in that study. Our study additionally took account of physical performance and found that the previous findings restricted to T2DM population could be extrapolated to community-dwelling settings regardless of T2DM history. A possible mechanism underlying such a relationship is that low GNRI can be a hallmark of malnutrition and systemic inflammation [52, 53], both greatly contributing to sarcopenia pathology [54, 55]. Under this hypothesis, stronger associations of GNRI with handgrip strength and muscle mass in the previous study [51] than in ours could be explained by more severe and longer duration of chronic inflammation in T2DM. We also found that ALB had a weaker correlation with muscle mass but a stronger correlation with muscle strength or physical performance than GNRI. However, ALB was not an independent indicator of sarcopenia risk after adjusting for confounders, which was contradictory to the finding in a previous meta-analysis that low ALB levels were significantly associated with the presence of sarcopenia in elderly adults [56]. The reason for disparity remains unclear but may lie in differences in ethnicity, settings, diagnostic criteria or age range between populations in the included studies and our study population.

Some anthropometric and nutritional risk-related indexes have also been previously investigated in sarcopenia [20–24]. CC has been reported to positively correlate with ASMI in both black women [20] and Japanese older adults [21]; Ling CHY et al. suggested that MAC and CC could be alternative measures for ASMI in elderly outpatients, while they showed weak associations with physical performance [22]. This was in agreement with our findings that CC and MAC were strongly associated with muscle mass but weakly associated with muscle strength or physical performance. Nishikawa H et al. found that TST had a positive correlation with ASMI in patients with liver diseases [23], while in our study with no restriction on comorbidities, TST was positively related to physical performance but negatively related to muscle strength with both weak associations, and there was no significant association between TST and ASMI. Yu R et al. showed that higher BMI was revealed to protect against sarcopenia incidence and reversibility [24]. Consistently, BMI in our study correlated strongly with muscle mass and weakly with muscle strength. Taken together, our findings may be explained by other potential determinants of muscle strength or physical performance in addition to muscle mass (e.g., upper extremity functional status, physical constructs) [25]. However, similar to GNRI, the four anthropometric indexes all turned to be independent risk factors for sarcopenia presence after adjusting for confounders, with the magnitude of their ORs parallel with the magnitude of the respective correlation coefficients. This suggested that sarcopenia diagnosis in our study population was more driven by muscle mass, possibly due to an unignorable proportion of people with low muscle strength (47.4%) or physical performance (38.3%). Inconsistent findings on the strengths of associations between previous studies and ours may be related to disparities involving race, settings, diagnostic standards, sample size, etc. For example, BMI was previously demonstrated to be a better predictor of sarcopenia than CC [57], which was contrary to the finding in our current study. A possible reason is that the former study was conducted in European nursing home residents, and sarcopenia was diagnosed according to the EWGSOP criteria [57]. It was also noteworthy that none of the concerned indexes except CC were significantly different between the severe and non-severe sarcopenia groups, indicating the potential value of CC in severity stratification for sarcopenia, which requires further investigation.

Regarding distinguishing between people with and without sarcopenia in our entire study population, diagnostic efficacy was the highest for MAC. A previous study based on the WCHAT study also suggested that MAC could serve as a proxy measure of ASMI with better performance than CC, but it adopted different exclusion criteria and thus included a different number of participants due to focusing solely on MAC and CC as diagnostic indexes [58]. MAC in our study also showed a high sensitivity at its optimal cut-off value, supporting its application as a screening instrument. GNRI, CC and BMI showed moderate diagnostic efficacy with nonsignificant differences. The discrimination ability of GNRI in our study was much higher than that in a previous study (AUC = 0.80 vs 0.68), which may be explained by European hospitalized patients (≥ 60 years) recruited and sarcopenia status defined by the EWGSOP algorithm in the previous study [38]. Although GNRI had an inferior AUC to MAC, it exhibited the highest accuracy and specificity at its optimal cut-off value. This indicated that GNRI may have the strongest classification ability to correctly detect and exclude patients of sarcopenia with the highest agreement rate, and it may also perform better for reducing misdiagnoses than missed diagnoses of sarcopenia but with potentially more false negative results. In contrast, CC showed the highest sensitivity, which was similar to that of MAC but a poor specificity at its optimal cut-off value, which verified its application for case-finding of sarcopenia as recommended by AWGS 2019 [9] and cautioned against false positives. We showed a moderate sensitivity, specificity and accuracy for BMI at the optimal cut-off value. BMI was previously shown to exert favorable effects on muscle mass and strength [24]; besides, a positive correlation has been observed between BMI and fat mass [24], the energy reserve whose levels may be proportional to protein intake, which can protect against sarcopenia [59, 60]. However, for overweight or obese people indicated by a relatively high BMI, their physical performance (e.g., gait speed) can be impaired [61, 62], which may weaken the value of BMI to detect sarcopenia. We did not show ideal diagnostic value of TST, the parameter to assess fat content and define energy reserve [18]. Consistently, TST in another study of patients with liver diseases performed poorly in sarcopenia diagnosis, despite a positive correlation of TST with ASMI [23]. A possible explanation is that internalizing and centralizing body fat is a common phenomenon in elderly people [63, 64], hampering the effectiveness of TST to evaluate nutrition status related to sarcopenia. We showed an even poorer diagnostic performance of ALB. There has been debate on the value of ALB in measuring nutrition status because its levels can be widely affected by various factors, such as different pathological states or drugs [17]. Utilization of ALB combined with weight and height can minimize confounding variables such as hydration status to some extent [26, 65], which may explain better diagnostic performance of GNRI than ALB. Taken together, for people with no access to standardized diagnostic modalities or advanced equipment in primary care settings, or with no desire for subsequent confirming procedures due to concerns about high cost or radiation exposure, a GNRI value below 108.1 may indicate a high chance of sarcopenia; in contrast, a CC value over 34.0 cm or MAC value over 27.5 cm may be better indicative of non-sarcopenia. Moreover, GNRI may also outperform MAC and CC considering possible errors of interpretation that can affect anthropometric measurements because adipose or connective tissues and edema can take the place of muscle tissues [18]. We further investigated diagnostic value of the concerned indexes to confirm the diagnosis in possible sarcopenia and yielded similar results, except that the specificity and accuracy of ALB at its optimal cut-off value greatly increased, suggesting a larger proportion of sarcopenia patients below the optimal cut-off value in possible sarcopenia than in the entire study population. This may likewise provide references on decision-making about whether to conduct DXA or BIA in patients suspected of having sarcopenia.

This study had several strengths. First, our data were derived from a community-dwelling elderly population of multiethnic backgrounds in China based on a relatively large sample size, which supported the generalization of our findings for middle-aged and elderly people in community-based settings. Second, we tried to include diagnostic indexes which were commonly adopted in nutritional assessment and available as many as possible, promoting the comprehensiveness and practical value of our study. We especially focused on GNRI, the index whose diagnostic utility to detect middle-aged and elderly adults with sarcopenia in Asian community-based settings has not been elucidated before. Third, the diagnostic procedures performed in the WCHAT study and our assessment or diagnosis related to sarcopenia were standardized and strictly in line with AWGS 2019 [9], which helped to strengthen the validity of our findings. Fourth, we controlled for as many available confounding variables as possible, which could better reflect the actual relationships we studied.

However, limitations in our study should also be noted. First, we excluded participants with unavailable, insufficient or missing information necessary for analysis as stated by exclusion criteria, which might introduce a potential selection bias. Second, participants recruited in the WCHAT study were residents in western China, which might limit the extrapolation of our findings to elderly people in Western countries, other Asian areas or other regions of China considering disparities in race, geography, lifestyles, etc. Third, we did not include some other nutritional risk-related indexes such as MNA-SF results in the final analysis due to large proportions of missing data. However, the MNA-SF is a subjective, questionnaire-based assessment tool [16] that is not applicable to elderly people with cognitive impairment or difficult to communicate with, thus laboratory or anthropometric indexes show advantages of being simpler and more objective. Moreover, this study has a cross-sectional design, so we expect more evidence from prospective clinical studies with good designs and large sample sizes to verify our findings and further clarify causal relationships or value of the indexes in sarcopenia risk prediction.

In conclusion, among these nutritional risk-related indexes whose application is simpler, less costly and better popularized in primary care, overall diagnostic performance was the best for MAC, followed by GNRI, CC, BMI, and the worst for TST, ALB in distinguishing sarcopenia from non-sarcopenia in middle-aged and elderly adults in community-based settings. CC or MAC might do better in reducing missed diagnosis, while GNRI was superior in reducing misdiagnosis with even better accuracy and fewer interpretation errors affecting anthropometric measurements.

## Supplementary Information

**Additional file 1: Supplementary Figure 1.** The correlogram showing reciprocal correlations of the potential variables to be controlled in model 3. The strength of correlations was represented by module color with a gradient from green (weakest) to red (strongest).

**Additional file 2: Supplementary Table 1.** The laboratory variables measured and analyzed.

**Additional file 3: Supplementary Table 2.** Comparison of baseline data between non-severe and severe sarcopenia.

**Additional file 4: Supplementary Table 3.** The variance inflation factors (VIF) for each potential continuous variable to enter model 3.

**Additional file 5: Supplementary Table 4.** Pairwise comparison on diagnostic measures of the concerned indexes in the entire study population.

**Additional file 6: Supplementary Table 5.** Pairwise comparison on diagnostic measures of the concerned indexes in possible sarcopenia.

## Data Availability

The datasets generated and/or analysed during the current study are not publicly available because the WCHAT study is still ongoing, but the relevant data are available from the corresponding author on reasonable request.
