# Supplementary figures and images for: Associations of geriatric nutrition risk index and other nutritional risk-related indexes with sarcopenia presence and their value in sarcopenia diagnosis
Source: BMC Geriatr. 2022 Apr 15;22:327. doi: 10.1186/s12877-022-03036-0 (PMC9012026; doi:10.1186/s12877-022-03036-0)

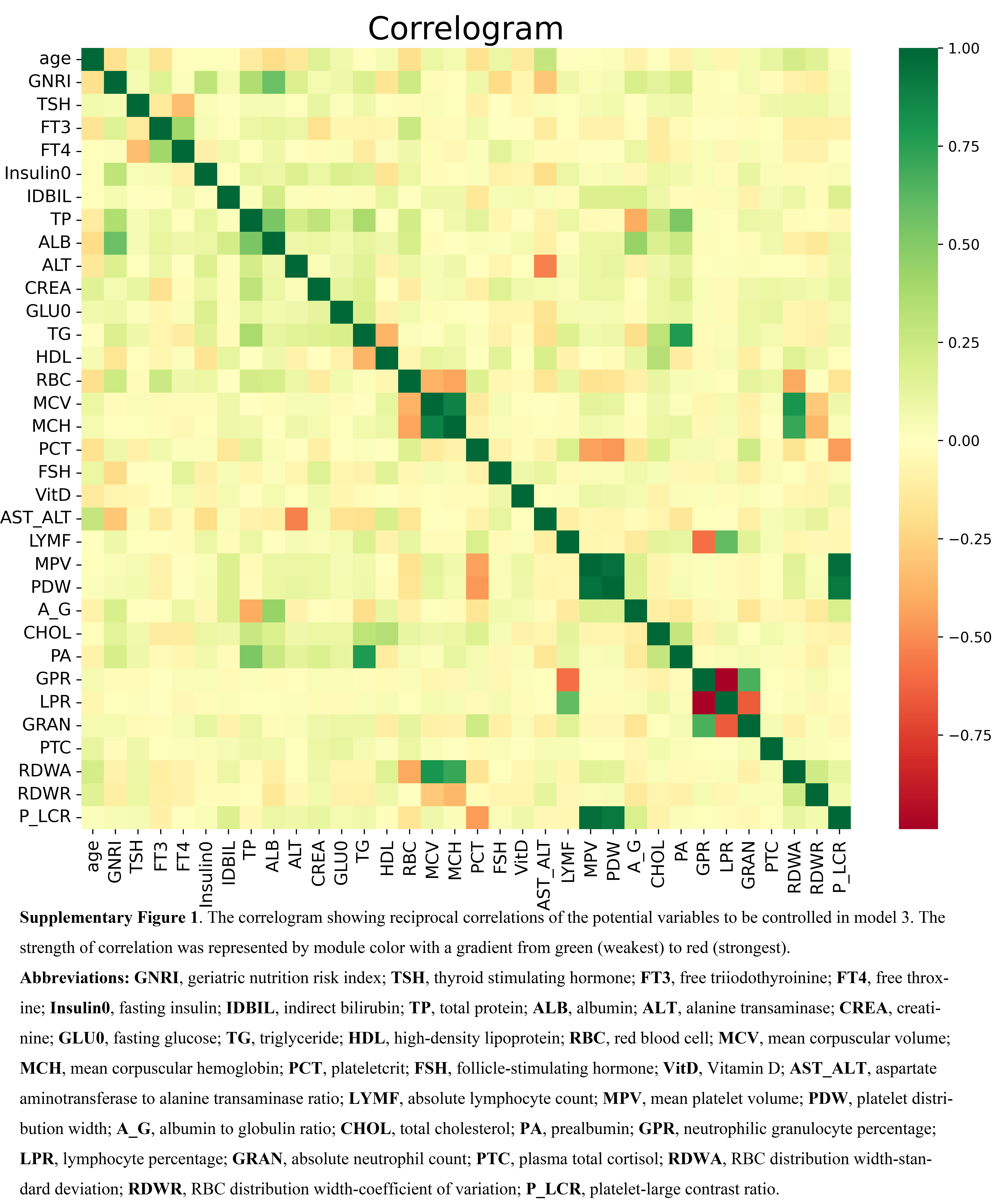

Supplement: Supplementary file 1 — Additional file 1: Supplementary Figure 1. The correlogram showing reciprocal correlations of the potential variables to be controlled in model 3. The strength of correlations was represented by module color with a gradient from green (weakest) to red (strongest). [file 12877_2022_3036_MOESM1_ESM.tif]
