# Supplementary material for: Associations of geriatric nutrition risk index and other nutritional risk-related indexes with sarcopenia presence and their value in sarcopenia diagnosis
Source: BMC Geriatr. 2022 Apr 15;22:327. doi: 10.1186/s12877-022-03036-0 (PMC9012026; doi:10.1186/s12877-022-03036-0)
Supplement: Supplementary file 2 — Additional file 2: Supplementary Table 1. The laboratory variables measured and analyzed. [file 12877_2022_3036_MOESM2_ESM.docx]

**Supplementary Table 1.** The laboratory variables measured and analyzed.

| **Variables** | **Abbreviations** |
| --- | --- |
| **Blood biochemical test** |  |
| Total bilirubin | / |
| Direct bilirubin | / |
| Indirect bilirubin | / |
| Total protein | / |
| Globulin | / |
| Alanine transaminase | ALT |
| Aspartate aminotransferase | AST |
| Creatinine | CREA |
| Urea | / |
| Uric acid | / |
| Glucose | GLU |
| Triglyceride | TG |
| Total cholesterol | TC |
| High-density lipoprotein | HDL |
| Low-density lipoprotein | LDL |
| **Blood routine test** |  |
| White blood cell | WBC |
| Absolute neutrophil count | / |
| Absolute lymphocyte count | / |
| Neutrophilic granulocyte percentage | / |
| Lymphocyte percentage | / |
| Red blood cell | RBC |
| RBC distribution width-standard deviation | RDW-SD |
| RBC distribution width-coefficient of variation | RDW-CV |
| Hemoglobin | / |
| Hematocrit | / |
| Mean corpuscular volume | / |
| Mean corpuscular hemoglobin | / |
| Mean corpuscular hemoglobin concentration | MCHC |
| Platetlet | / |
| Plateletcrit | / |
| Mean platelet volume | / |
| Platelet distribution width | / |
| Platelet large cell ratio | / |
| **Other variables** |  |
| Thyroid stimulating hormone | / |
| Free triiodothyroinine | FT3 |
| Free throxine | FT4 |
| Fasting insulin | INS |
| Plasma total cortisol; | / |
| Vitamin D | VitD |
