# Supplementary material for: Associations of geriatric nutrition risk index and other nutritional risk-related indexes with sarcopenia presence and their value in sarcopenia diagnosis
Source: BMC Geriatr. 2022 Apr 15;22:327. doi: 10.1186/s12877-022-03036-0 (PMC9012026; doi:10.1186/s12877-022-03036-0)
Supplement: Supplementary file 3 — Additional file 3: Supplementary Table 2. Comparison of baseline data between non-severe and severe sarcopenia. [file 12877_2022_3036_MOESM3_ESM.docx]

**Supplementary Table 2.** Comparison of baseline data between non-severe and severe sarcopenia.

|  | **Non-severe Sarcopenia**  **n=261** | **Severe Sarcopenia**  **n=255** | **P value** |
| --- | --- | --- | --- |
| **Age** | 66.00 [61.00,73.00] | 72.00 [66.00,77.00] | <0.001 |
| **Sex, n (%)** |  |  | 0.227 |
| Male | 155 (59.39) | 137 (53.73) |  |
| Female | 106 (40.61) | 118 (46.27) |  |
| **Ethnicity, n (%)** |  |  | 0.767 |
| Han | 136 (52.11) | 125 (49.02) |  |
| Qiang | 41 (15.71) | 39 (15.29) |  |
| Tibetan | 58 (22.22) | 64 (25.10) |  |
| Yi | 23 (8.81) | 21 (8.24) |  |
| Other minority | 3 (1.15) | 6 (2.35) |  |
| **Marital status, n (%)** |  |  | 0.003 |
| Single | 5 (1.92) | 3 (1.18) |  |
| Married | 215 (82.38) | 177 (69.41) |  |
| Divorced | 4 (1.53) | 6 (2.35) |  |
| Widowed | 37 (14.18) | 69 (27.06) |  |
| **Smoking, n (%)** | 81 (31.03) | 69 (27.06) | 0.37 |
| **Drinking alcohol, n (%)** | 64 (24.52) | 72 (28.24) | 0.391 |
| **ADL, n (%)** |  |  | 0.037 |
| Normal ADL | 231 (88.51) | 208 (81.57) |  |
| ADL impairment | 30 (11.49) | 47 (18.43) |  |
| **IADL, n (%)** |  |  | <0.001 |
| Normal IADL | 193 (73.95) | 143 (56.08) |  |
| IADL impairment | 68 (26.05) | 112 (43.92) |  |
| **Moderate to severe cognitive impairment, n (%)** | 46 (17.62) | 62 (24.31) | 0.079 |
| **Number of comorbidities, n (%)** |  |  | 0.011 |
| 0 | 161 (61.69) | 127 (49.80) |  |
| 1 | 50 (19.16) | 53 (20.78) |  |
| ≥2 | 50 (19.16) | 75 (29.41) |  |
| **Moderate to severe anxiety, n (%)** | 7 (2.68) | 12 (4.71) | 0.324 |
| **Moderate to severe depression, n (%)** | 11 (4.21) | 17 (6.67) | 0.301 |
| **Total bilirubin (umol/L)** | 17.70 [13.80,20.30] | 18.30 [14.30,20.40] | 0.528 |
| **Direct bilirubin (umol/L)** | 5.50 [4.30,7.50] | 5.40 [4.40,7.10] | 0.86 |
| **Indirect bilirubin (umol/L)** | 11.60 [9.30,14.30] | 12.20 [9.55,14.00] | 0.565 |
| **Total protein (g/L)** | 70.64 (4.99) | 70.59 (5.27) | 0.906 |
| **Globulin (g/L)** | 27.42 (3.95) | 27.76 (4.02) | 0.323 |
| **ALT (U/L)** | 20.00 [16.00,29.00] | 19.00 [14.00,25.00] | 0.02 |
| **AST (U/L)** | 27.00 [23.00,33.00] | 27.00 [23.00,32.50] | 0.882 |
| **CREA (umol/L)** | 81.96 (17.49) | 84.35 (22.21) | 0.175 |
| **Urea (mmol/L)** | 5.52 (1.58) | 5.48 (2.05) | 0.802 |
| **Uric acid (umol/L)** | 325.89 (89.50) | 333.17 (91.31) | 0.361 |
| **GLU (mmol/L)** | 4.97 [4.66,5.42] | 5.08 [4.69,5.53] | 0.296 |
| **TG (mmol/L)** | 1.22 [0.89,1.76] | 1.23 [0.88,1.63] | 0.56 |
| **TC (mmol/L)** | 4.61 (0.85) | 4.72 (1.05) | 0.196 |
| **HDL (mmol/L)** | 1.30 (0.33) | 1.38 (0.38) | 0.013 |
| **LDL (mmol/L)** | 2.61 (0.74) | 2.69 (0.85) | 0.273 |
| **WBC (10^9^/L)** | 5.60 [4.90,6.70] | 5.80 [4.80,6.70] | 0.926 |
| **Absolute neutrophil count (10^9^/L)** | 3.50 [2.70,4.30] | 3.60 [2.70,4.35] | 0.993 |
| **Absolute lymphocyte count (10^9^/L)** | 1.60 [1.40,2.00] | 1.60 [1.30,2.00] | 0.373 |
| **Neutrophilic granulocyte percentage (%)** | 61.98 (9.89) | 62.69 (9.04) | 0.396 |
| **Lymphocyte percentage (%)** | 30.67 (8.90) | 30.10 (8.35) | 0.451 |
| **RBC (10^12^/L)** | 4.88 (0.59) | 4.83 (0.65) | 0.356 |
| **RDW-SD (fL)** | 53.80 (4.94) | 53.87 (5.31) | 0.879 |
| **RDW-CV (%)** | 14.83 (0.83) | 15.05 (1.04) | 0.008 |
| **Hemoglobin (g/L)** | 149.10 (18.06) | 146.40 (19.19) | 0.1 |
| **Hematocrit (L/L)** | 47.53 (6.10) | 46.64 (6.31) | 0.104 |
| **Mean corpuscular volume (fL)** | 97.30 [94.20,100.00] | 96.80 [93.60,99.80] | 0.318 |
| **Mean corpuscular hemoglobin (pg)** | 30.70 [29.60,31.70] | 30.50 [29.60,31.40] | 0.315 |
| **MCHC (g/L)** | 315.00 [311.00,319.00] | 315.00 [311.00,320.00] | 0.849 |
| **Platetlet (10^9^/L)** | 169.11 (59.14) | 168.47 (56.19) | 0.901 |
| **Plateletcrit (%)** | 0.17 [0.14,0.21] | 0.18 [0.14,0.20] | 0.988 |
| **Mean platelet volume (fL)** | 10.70 [9.70,12.10] | 10.70 [9.70,11.85] | 0.965 |
| **Platelet distribution width (%)** | 13.10 [11.70,14.80] | 13.00 [11.70,14.50] | 0.787 |
| **Platelet large cell ratio (%)** | 32.10 [24.30,41.20] | 31.50 [25.15,40.00] | 0.798 |
| **Thyroid stimulating hormone (mU/L)** | 2.68 [1.66,4.31] | 2.62 [1.77,4.01] | 0.71 |
| **FT3 (pmol/l)** | 4.49 [4.06,4.89] | 4.25 [3.86,4.65] | <0.001 |
| **FT4 (pmol/l)** | 18.41 [16.55,20.49] | 18.45 [16.20,20.51] | 0.812 |
| **INS (uU/ml)** | 4.43 [3.01,6.35] | 4.74 [3.13,6.89] | 0.604 |
| **Plasma total cortisol (nmol/L)** | 377.76 (130.68) | 389.09 (141.34) | 0.345 |
| **VitD (ng/ml)** | 18.80 (5.82) | 18.36 (6.90) | 0.439 |
| **GNRI** | 105.20 (7.83) | 104.71 (8.51) | 0.492 |
| **ALB (g/L)** | 43.22 (3.11) | 42.82 (3.54) | 0.173 |
| **CC (cm)** | 31.99 (2.77) | 31.40 (2.74) | 0.016 |
| **MAC (cm)** | 25.57 (2.46) | 25.44 (2.78) | 0.575 |
| **TST (cm)** | 18.33 (7.00) | 18.78 (7.55) | 0.488 |
| **BMI (kg/m^2^)** | 21.88 (2.99) | 21.95 (3.04) | 0.79 |
| **ASMI (kg/m^2^)** | 5.87 (0.78) | 5.71 (0.84) | 0.029 |
| **Handgrip strength (kg)** | 20.28 (7.32) | 16.14 (5.65) | <0.001 |
| **Time consumed in the 4-meter walking test (s)** | 5.27 (1.81) | 6.61 (3.62) | <0.001 |
| **Time consumed in the 5-time chair stand test (s)** | 11.16 (2.96) | 15.46 (3.52) | <0.001 |

**Note**: data were presented as mean (standard deviation), median [quartile 1, quartile 3] or n (%) as appropriate.

**Abbreviations**: **ADL**, Activities of Daily Living; **IADL,** Instrumental ADL; **ALT**, alanine transaminase; **AST** aspartate aminotransferase; **CREA**, creatinine; **GLU**, glucose; **TG**, triglyceride; **TC,** total cholesterol; **HDL**, high-density lipoprotein; **LDL,** low-density lipoprotein**; WBC,** white blood cell; **RBC**, red blood cell; **RDW-SD,** RBC distribution width-standard deviation; **RDW-CV,** RBC distribution width-coefficient of variation; **MCHC**, mean corpuscular hemoglobin concentration; **FT3**, free triiodothyroinine; **FT4**, free throxine; **INS**, fasting insulin; **VitD**, Vitamin D; **GNRI**, geriatric nutrition risk index; **ALB**, albumin; **CC**, calf circumference; **MAC,** mid-arm circumference; **TST**, triceps skinfold thickness; **BMI**, body mass index; **ASMI**, appendicular skeletal muscle mass index.
