# Supplementary material for: Associations of geriatric nutrition risk index and other nutritional risk-related indexes with sarcopenia presence and their value in sarcopenia diagnosis
Source: BMC Geriatr. 2022 Apr 15;22:327. doi: 10.1186/s12877-022-03036-0 (PMC9012026; doi:10.1186/s12877-022-03036-0)
Supplement: Supplementary file 4 — Additional file 4: Supplementary Table 3. The variance inflation factors (VIF) for each potential continuous variable to enter model 3. [file 12877_2022_3036_MOESM4_ESM.docx]

**Supplementary Table 3.** The variance inflation factors (VIF) for each potential continuous variable to enter model 3.

| **Variables** | **VIFs** |
| --- | --- |
| **Lymphocyte percentage** | 55.95523 |
| **Neutrophilic granulocyte percentage** | 52.05709 |
| **Mean platelet volume** | 23.05758 |
| **Platelet large cell ratio** | 15.00453 |
| **Platelet distribution width** | 10.64852 |
| **RDW-SD** | 10.19071 |
| **Mean corpuscular volume** | 9.019463 |
| **Absolute neutrophil count** | 8.737321 |
| **Total protein** | 8.287132 |
| **Absolute lymphocyte count** | 7.890975 |
| **Mean corpuscular hemoglobin** | 6.556198 |
| **TG** | 4.183331 |
| **RDW-CV** | 4.148258 |
| **Prealbumin** | 3.771256 |
| **HDL** | 1.980625 |
| **TC** | 1.80556 |
| **RBC** | 1.666987 |
| **Plateletcrit** | 1.56782 |
| **ALT** | 1.5346 |
| **CREA** | 1.524023 |
| **FT4** | 1.466176 |
| **FT3** | 1.451002 |
| **Age** | 1.341998 |
| **INS** | 1.213555 |
| **Thyroid stimulating hormone** | 1.197986 |
| **GLU** | 1.164065 |

**Abbreviations**: **ALT**, alanine transaminase; **CREA**, creatinine; **GLU**, glucose; **TG**, triglyceride; **TC,** total cholesterol; **HDL**, high-density lipoprotein; **RBC**, red blood cell; **RDW-SD,** RBC distribution width-standard deviation; **RDW-CV,** RBC distribution width-coefficient of variation; **FT3**, free triiodothyroinine; **FT4**, free throxine; **INS**, fasting insulin.
