# Supplementary material for: Associations of geriatric nutrition risk index and other nutritional risk-related indexes with sarcopenia presence and their value in sarcopenia diagnosis
Source: BMC Geriatr. 2022 Apr 15;22:327. doi: 10.1186/s12877-022-03036-0 (PMC9012026; doi:10.1186/s12877-022-03036-0)
Supplement: Supplementary file 6 — Additional file 6: Supplementary Table 5. Pairwise comparison on diagnostic measures of the concerned indexes in possible sarcopenia. [file 12877_2022_3036_MOESM6_ESM.docx]

**Supplementary Table 5.** Pairwise comparison on diagnostic measures of the concerned indexes in possible sarcopenia.

| **Pairwise comparison on diagnostic measures** | **P value** |
| --- | --- |
| **AUC** |  |
| GNRI vs ALB | <0.001 |
| GNRI vs CC | 0.176 |
| GNRI vs MAC | <0.001 |
| GNRI vs TST | <0.001 |
| GNRI vs BMI | 0.002 |
| ALB vs CC | <0.001 |
| ALB vs MAC | <0.001 |
| ALB vs TST | <0.001 |
| ALB vs BMI | <0.001 |
| CC vs MAC | 0.006 |
| CC vs TST | <0.001 |
| CC vs BMI | 0.635 |
| MAC vs TST | <0.001 |
| MAC vs BMI | 0.018 |
| TST vs BMI | <0.001 |
|  |  |
| **Sensitivity** |  |
| GNRI vs MAC | <0.001 |
| GNR vs CC | <0.001 |
| GNRI vs TST | 0.554 |
| GNRI vs ALB | <0.001 |
| GNRI vs BMI | 0.006 |
| MAC vs CC | 0.197 |
| MAC vs TST | <0.001 |
| MAC vs ALB | <0.001 |
| MAC vs BMI | 0.031 |
| CC vs TST | <0.001 |
| CC vs ALB | <0.001 |
| CC vs BMI | 0.001 |
| TST vs ALB | <0.001 |
| TST vs BMI | 0.001 |
| ALB vs BMI | <0.001 |
|  |  |
| **Specificity** |  |
| GNRI vs MAC | <0.001 |
| GNR vs CC | <0.001 |
| GNRI vs TST | <0.001 |
| GNRI vs ALB | 0.101 |
| GNRI vs BMI | <0.001 |
| MAC vs CC | <0.001 |
| MAC vs TST | <0.001 |
| MAC vs ALB | <0.001 |
| MAC vs BMI | 0.888 |
| CC vs TST | 0.384 |
| CC vs ALB | <0.001 |
| CC vs BMI | <0.001 |
| TST vs ALB | <0.001 |
| TST vs BMI | <0.001 |
| ALB vs BMI | <0.001 |
|  |  |
| **Accuracy** |  |
| GNRI vs MAC | 0.002 |
| GNR vs CC | <0.001 |
| GNRI vs TST | <0.001 |
| GNRI vs ALB | 0.013 |
| GNRI vs BMI | <0.001 |
| MAC vs CC | <0.001 |
| MAC vs TST | <0.001 |
| MAC vs ALB | 0.561 |
| MAC vs BMI | 0.529 |
| CC vs TST | 0.002 |
| CC vs ALB | <0.001 |
| CC vs BMI | 0.001 |
| TST vs ALB | <0.001 |
| TST vs BMI | <0.001 |
| ALB vs BMI | 0.226 |

**Note**: The AUC was compared as proposed by DeLong et al^43^. The sensitivity, specificity or accuracy at the optimal cut-off value was compared with the McNemar chi-square test.

**Abbreviations**: **AUC**, area under the receiver operating characteristic curve; **GNRI**, geriatric nutrition risk index; **ALB**, albumin; **CC**, calf circumference; **MAC,** mid-arm circumference; **TST**, triceps skinfold thickness; **BMI**, body mass index.
